# Putting reasons back into reasoning: how genuine reasoning is inference-based and why neuro-symbolic NLI could achieve it

**DOI:** 10.3389/frai.2026.1801094

**Published:** 2026-05-08

**Authors:** Reto Gubelmann

**Affiliations:** Universität Zürich, Zürich, Switzerland

**Keywords:** inference, inferentialism, Leibniz, LLMs, neuro-symbolic NLP, reasoning, Natural Language Infernce

## Abstract

Taking Leibniz' ideal of a universal truth-calculating machine as a vantage point, this article provides a philosophically sound analysis of the concept of reasoning in NLP. It argues that reasoning always involves inference, which in turn requires being guided by reason relations. Based on this, the article argues that Symbolic NLP is unable to reason for epistemic reasons on the part of humans, that Neural NLP is likely unable to do so in principle, and that Neuro-Symbolic NLP is ideally set up to succeed where the two approaches in isolation failed, that is, to progress toward realizing Leibniz' vision of a truth-calculating machine—to the extent to which this is possible. We conclude by providing a theoretical grounding for the latter claim in the philosophy of a contemporary rationalist, namely Robert Brandom.

## 1 Introduction

In 1666, quite exactly 360 years ago from the year of publication of this article, a German philosopher and polymath published a dissertation called “Dissertation on the Art of Combinations” [“Dissertatio de arte combinatoria”, see (Look 2020) for an introduction to this text]. The name of the author is Gottfried Willhelm Leibniz, and he might have been one of the last polymaths of the West, who among many other things co-invented the differential calculus (Arthur and Rabouin, 2025), pioneered silk production to fund academic institutions (Bredekamp, 2012), and invented a breathtakingly innovative metaphysics of mind and body (Look, 2020).

In this article, however, we focus on another vision that he sketched in his dissertation and continued to pursue long after: The often slightly ridiculed idea of the so-called *calculus ratiocinator*, a universal truth-calculation machine (called *calculus* in the following). Its basic idea was to build an algorithm, or a machine to literally compute the truth value of any proposition whatsoever, just like one can compute the result of any arithmetic operation. To do this, Leibniz conceived an highly innovative intensional logic inspired by the Aristotelian syllogistic that was state of the art at the time [see (Lenzen 2018) for an introduction]. Furthermore, Leibniz thought that the calculus required a universal, non-ambiguous, highly systematic language [much like what the early Wittgenstein (2006–1922) and (Carnap 1999) attempted in the 20th century], what he called a *characteristica universalis*. This ideal language would be essential for the *calculus* to derive the truth value of any proposition with mathematical precision and mechanistic objectivity. Once this calculus was available, when a disagreement on the truth of any claim would occur, one would then no more have to discuss potentially endlessly, but one could just calculate—hence his slogan: *calculemus*—Let's calculate!

**Figure 1:**
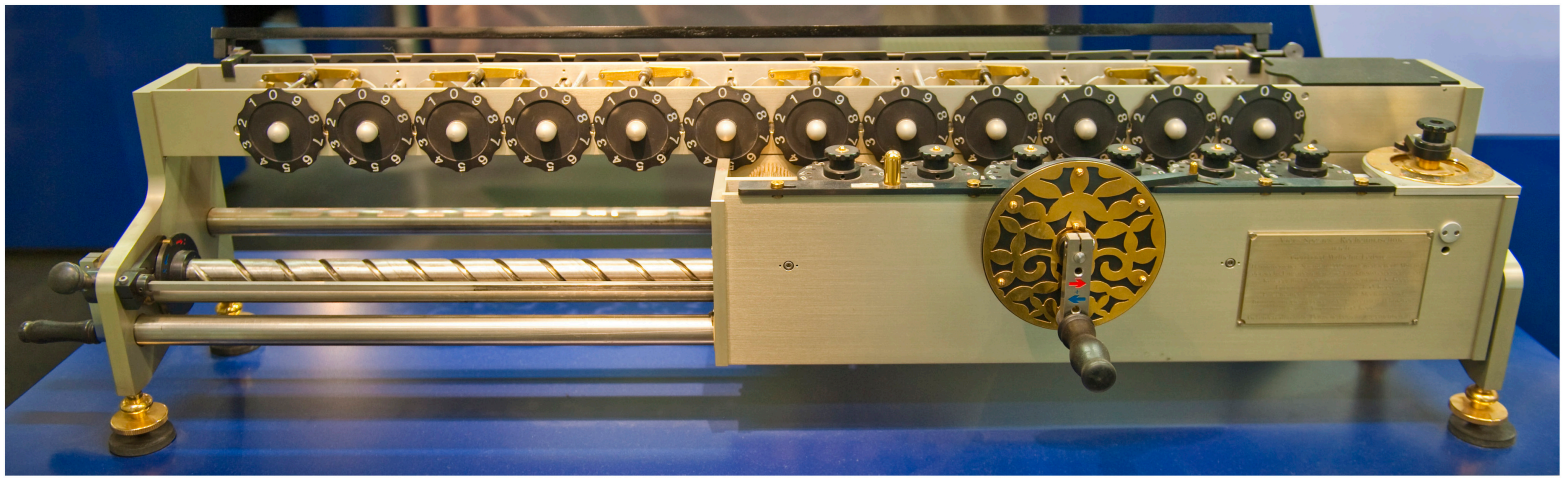
The arithmetic calculator invented by Leibniz, being the first calculator to master all of the four elementary operations of addition, subtraction, multiplication, and division—Leibniz envisaged a similar machine to assess the truth of any claim (Picture: Hannes Grobe, CC Attribution 3.0).

The word that Leibniz chose—*ratiocinator*—perfectly expresses his ambition: Its root, the verb *ratiocinari*, can mean calculating (for instance in bookkeeping), but it can also mean reasoning or inferring. This embodies Leibniz's dream: To transform reasoning into an objective, implementable process of calculation. Indeed, Leibniz literally envisaged machines that could execute his calculus, similar to the one he developed for arithmetic operations, see Figure 1.

Before considering the current state of Natural Language Processing (NLP), it is worth revisiting pre-NLP intellectual achievements that moved the needle forward to something like Leibniz' universal calculus: The development of modern sound and complete logical calculi, as pioneered by the Begriffsschrift of (Frege 1879). Following his lead, 20th century logicians developed ever more efficient and powerful calculi, perhaps culminating in modal logical calculi championed by Kripke (1983/1963) and (Plantinga 1974), the latter notably sharing an interest in ontological arguments with Leibniz. By the same token, research from the same area showed some principled limitations to Leibniz' vision of the universal calculus, when cashed out using first-order logic: The results obtained by (Church 1936) and (Turing 1936) entail that, to put a complex matter rather simply, first-order logic is semi-decidable: while we can algorithmically enumerate all logical truths, there is no algorithm that can consistently identify non-validities or determine the truth value of any arbitrary proposition. See (Deutsch and Marshall 2025) for an introduction, and (Ben-Ari 2012) for a recent, computer-science-focused exposition of these results.^1^

In combination, these pre-NLP developments mean that it is now in principle possible to compute the truth value of most, albeit not all, claimed logical truths, given that they are expressed in a suitable formal language.^2^ In this way, we could still reliably compute inferences within the depth and complexity typical of human natural language discourse. As a consequence of the results obtained by Church and Turing, this would not be a universal calculus in the sense of being able to prove any logical truth whatsoever. However, we submit that it could still be a pragmatically universal calculus, that is, a calculus that is going to be able to prove the vast majority of first-order logical truths that can reasonably be expected to having originated from real-world natural language discourse.

What remained elusive, however, was the reliable automatic translation of natural language into such formal languages as first-order logic. Leibniz foresaw the difficulty with postulating his *characteristica universalis*, but this did not help in practice: How are we to deal with the complex, ambiguous and often seemingly chaotic nature of natural language?

Enter Natural Language Processing (NLP). Here, “reasoning” is currently a very prominent term. From the very established task of common-sense reasoning [compare (Brewka 1991) for a classical introduction] over a number of architectures and methods claiming to have finally mastered system 2 reasoning (Yang et al., 2025) to the currently popular “reasoning LLMs” (Besta et al., 2025), the term is used widely and prominently. Indeed, (Ke et al. 2025) list over 60 thousand papers published between 2022 and February 2025 dedicated in some way to reasoning and LLMs. Furthermore, in Language AI,^3^ there is currently a lively discussion around the questions of whether a purely neural, likely transformer-based (Vaswani et al., 2017) approach can deliver truly generalizable and reliable reasoning, or whether a Neuro-Symbolic approach is needed. In this discussion, we focus on a subfield of NLP, called “Natural Language Inference” (NLI) whose goal is to accurately recognize the logical relationship between two claims. Solving NLI would be tantamount to realizing Leibniz' vision for the restricted field of logical truths within the known principled limits.

In this field, our article makes two main contributions. (1) We propose a philosophically sound analysis of the concept of reasoning in NLP, which is inspired by a contemporary school of thought in philosophy of language called inferentialism, as pioneered by Robert Brandom. We argue that (2) there are empirical reasons (stemming from a survey of the state of the field) and theoretical reasons (based on an inferentialist conception of language) that suggest that both a purely symbolic and a purely neural approach to NLI will reach a glass ceiling, while a Neuro-Symbolic approach could succeed, indeed, that proposals in this general methodology, especially at the task of autoformalization, have matured to a degree that ground optimism regarding the realization of a pragmatically universal calculus.

Our argument proceeds as follows. We first analyze the concepts of *reasoning* and *inference*, as used in contemporary NLP, arguing that reasoning presupposes inferring, where inferring is understood as being guided by reason relations. Then, we argue that empirical research in NLP of the past decades shows that (1) purely symbolic approaches fail because humans are unable to make explicit all the implicit linguistic knowledge that they have, (2) purely neural approaches do not deliver genuine reasoning, but at most smart guessing, and (3) the combination of the two—Neuro-Symbolic Methods—is ideally set up to meet both challenges. Finally, we ground this empirical observation in a cutting-edge view in philosophy of language, called inferentialism, which also allows us to see how Leibniz' vision of a *characteristica universalis* can be realized in natural language, when properly systematized and explicated into a formal representation.

## 2 Genuine reasoning in language AI: drawing the conceptual map

In this section, we will chart the conceptual landscape surrounding reasoning in NLP. We will suggest that reasoning essentially involves inferring, where inferring is understood as proceeding along, and because of reason relations. We begin by delineating our notion of inference (Section 2.1), then, we argue why reasoning should be conceived as involving inference, so conceived, and we use this to develop a systematic of the current uses of reasoning in NLP (Section 2.2).

### 2.1 Theoretical grounding: to infer is to be guided by reasons

We rely on an established understanding of the concept of inference in the philosophy of language and logic, where inference means proceeding from (hypothetical or actual) acceptance of one proposition, or claim, to acceptance of another because one takes acceptance of the former to be a good reason for acceptance of the latter [see (Hlobil and Brandom 2024) for a recent, book-length exposition of the notion of inference along these lines]. This way, we cash out Leibniz' core vision of calculating truths, slightly anachronistically put: The resulting verdict—true or false—should be a computation from the truth values of the constituent propositions and their connection via truth-functional connectives. Any other cause for a verdict, say, the number of characters in the premises, would run counter Leibniz' vision of calculating truth in a objective, algorithmic way.

#### 2.1.1 Varieties of inference

This understanding of inference allows for a variety of kinds of inferences, in particular of formal-deductive inferences, which are valid or invalid based on the form of the propositions involved, as opposed to material inferences, which are valid or invalid due to the conceptual material involved. Compare Example (1) for a formally valid inference and (2) for a materially valid inference.

(1) All panthers are mammals, and all mammals are mortal, therefore, all panthers are mortal.

(2) Berlin is south of Oslo; therefore, Oslo is north of Berlin.

Furthermore, unlike in the case of formally valid inferences, the validity of materially valid inferences can be defeasible, see Example (3), and/or perspectival, see Example (4).

(3) Scott is a polar bear, whereas Jenny is a gorilla; therefore, Scott is more likely to survive a winter in the arctic.

(4) Sarah has been flying 7 hours to Bali for a week-end Yoga retreat; therefore, Sarah has acted immorally on that week-end.

Example (2) (as well as Example (1)) is deductively valid. This means that the truth of the premise necessitates the truth of the conclusion; framed in the vocabulary of monotonicity: No matter how many other premises you add, the inference remains valid. In contrast, Example (3) contains an inductive, defeasible, or non-monotonic inference: as it stands, it seems reasonable to infer the conclusion considering the reason given, but the inference is fallible. By sheer coincidence, the bear could die while the Gorilla survives, or there might be further information that subverts the validity of the inference. For instance, we might learn that the bear is terminally ill, while the Gorilla will be kept at an arctic research facility to study the impact of the darkness on Gorilla psychology, while making every effort that she survives.

Furthermore, the validity of inferences such as Example (4) depends on the perspective of the subject judging the inference. If her overall worldview is strongly hedonistic, she might fiercely dispute the validity of the inference, whereas a subject that is highly focused on reducing her carbon footprint for the greater good of the planet might, with the same conviction, underwrite the validity of the inference. With formally valid inferences, neither of the two phenomena are possible: All formally valid inferences are deductively valid, and the only perspective one needs to adopt to determine their validity is one of a competent, perhaps logically schooled speaker of the language in question.

#### 2.1.2 Along vs. because of reason relations: system 1 vs. system 2

We emphasize that genuine inference requires that one processes from premise to hypothesis because the premise is taken to be a good reason for the hypothesis. Hence, the reason relation must be the decisive causal factor in proceeding from acceptance of premise to acceptance of hypothesis.

This means that reliability per se is orthogonal to the question of whether or not a certain system operates inferentially. Let us assume that a certain dataset contains 400k premise-hypothesis pairs. And let us further assume that (1) all and only the pairs contradicting each other contain a negation in the premise, but not in the hypothesis, and (2) an encoder-only transformer such as BERT (Devlin et al., 2019) is fine-tuned on the training dataset and generalizes along this artifact of the dataset. It will then perform with the exact same reliability on that dataset regarding contradictions as a human being would that has mastered the actual concept of contradiction rather than the shallow heuristic of saying contradiction whenever only the premise contains a negation. However, we would not want to say that BERT draws a genuine inference; rather, it uses a shallow heuristic: never mind the question whether the premise is indeed contradicting the conclusion—indeed, just check whether only the premise is negated.^4^

This directly connects to a way of thinking about reasoning that has been popularized in psychology and economics, namely the so-called dual process theory of cognition proposed by (Tversky and Kahneman 1974); (Kahneman 2003); (Kahneman et al. 2021), which is in NLP usually referred to as system 1 vs. system 2 thinking. According to it, humans have two different processes, or systems of thinking about a subject matter. The first one is the older one, it is fast and associative, relying on heuristics and biases.^5^ The second one, in contrast, is evolutionarily more recent, much slower, and it allows humans to take a step back and reflect on the subject matter in a logical way not guided by biases and heuristics (see also Kogler and Khberger, 2007; Evans and Curtis-Holmes, 2005; Frankish, 2010). Given that reasoning necessarily involves inferring, we submit that system 1 thinking simply does not count as reasoning: It's what you do when you lack the time or the information to reason, it has its clear survival value, but it should not count as inference-based ratiocination. It's guessing rather than reasoning.

Finally, we note that the conception of inference proposed here differs from extremely wide understandings of the term, as used by some psychologists, e.g.: “the production of new mental representations on the basis of previously held representations” (Mercier and Sperber, 2011). On this understanding of inference, any kind of systematic association between two representations would be considered inference. For example, imagine that you have been drinking many Mojitos when you were on vacation in Miami Beach. Then, it is likely that the taste of a Mojito brings back vivid memories of the blue color of the ocean, of the salty air, etc. However, it seems inappropriate to say that you infer the blueness of the ocean from the taste of the Mojito.

#### 2.1.3 “Running inference” with an LLM

Finally, there is another meaning of inference currently popular in NLP: That of running inference with an LLM. In the community, “running inference” is opposed to training an LLM: While the latter involves optimizing the LLM's weights on a certain dataset, the former refers to forward-passes in an LLM, leading to autoregressive prediction of one token after another. This constitutes a particularly complex use of “inference”. On the one hand, it constitutes a textbook and, as the success of LLMs testifies, very powerful case of a probabilistic process, sometimes called a probabilistic inference: given the previously generated tokens and the weights of the LLM as well as the temperature hyperparameter, the LLM generates the next token. It is accurate to say that it proceeds to generate the next token based on its weights and the previous tokens. However, this should not be confused with any propositional inference as understood here. For instance (and rather famously), while adding a negation to a premise might completely invert the inferential relationship between premise and hypothesis, it might make a marginal difference to the LLM's probabilistic process of generating the next token (Gubelmann and Handschuh, 2022). So, it is a *bona fide* probabilistic prediction, sometimes called inference, but it is not a proceeding from accepting one proposition to accepting another proposition because one takes the former to be a reason for the latter.

**Figure 2:**
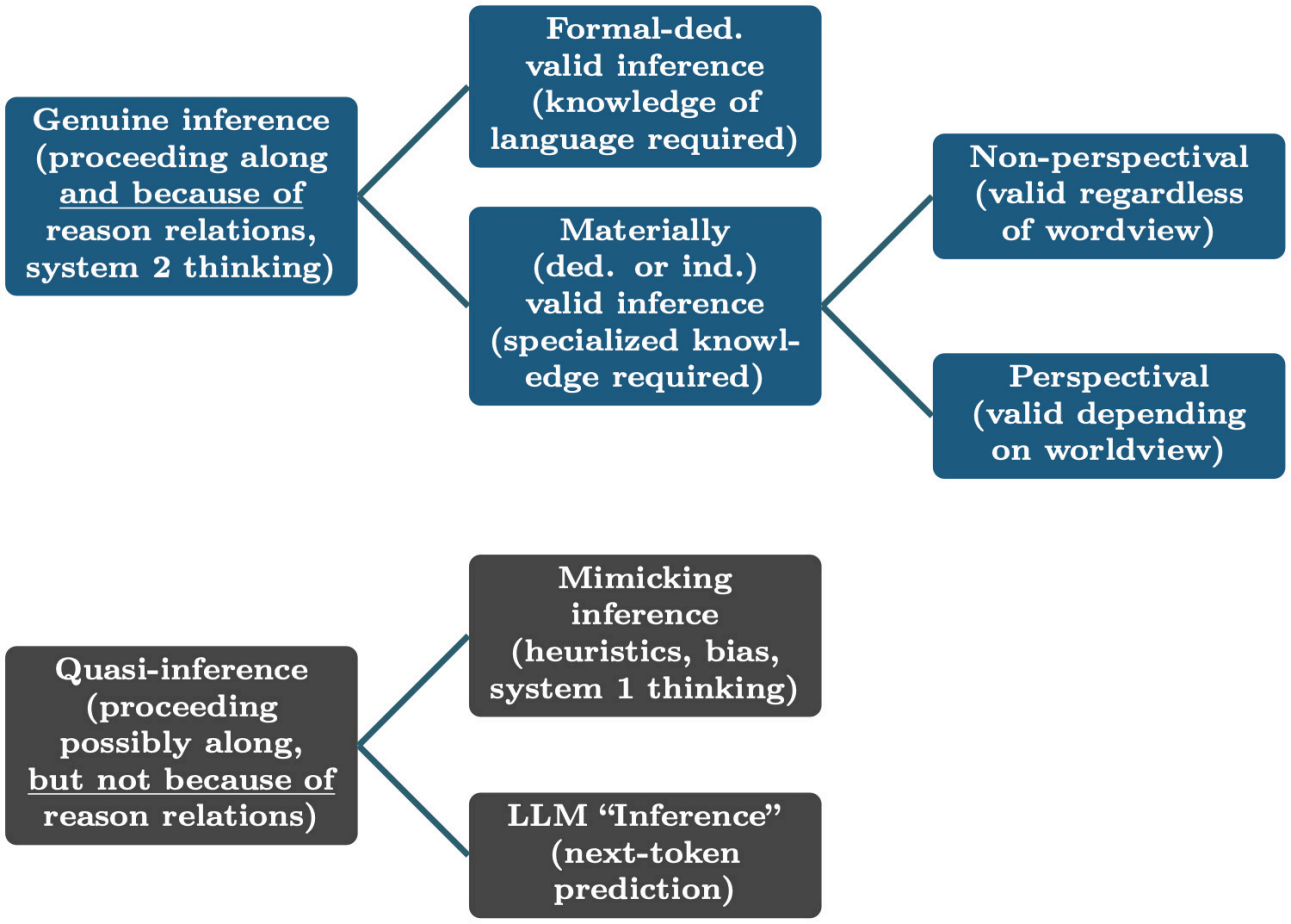
Overview on kinds of inference and quasi-inference, as relevant to NLP/Language AI.

Figure 2 gives an overview on this systematic of the uses of the concept of inference. We distinguish between genuine inferences and quasi-inferences. Genuine inferences consist in proceeding from the acceptance of a proposition, called the premise, to the acceptance of another proposition, called the conclusion, because on takes the former to be a reason for the latter. They come in two main kinds, formal and material; the material ones can be deductively or inductively valid, while all formally valid inferences are deductively valid. Among material inferences, there are non-perspectival and perspectival ones. Quasi-inferences are proceeding from a premise to a conclusion that is possibly along reason relations but not because of reason relations.

### 2.2 Delineating reasoning and quasi-reasoning in NLP

As mentioned above, “reasoning” is currently a highly sought-after label for NLP systems. And as it is with such high-value labels, there is a tendency to overuse it. In this section, we suggest that genuine reasoning requires genuine inference, as developed in the preceding section, that is, transfer of acceptance of one proposition, the premise, to acceptance of another proposition, the conclusion, *because the premise is taken to be a good reason for the conclusion*. We suggest that genuine reasoning always involves genuine inference.

In NLP, doing so has a number of advantages; we explicitly mention three of them. Perhaps most importantly, it agrees with established usage of the term in ordinary language and linguistic and philosophical theory: To reach a verdict based on reasoning means to reach it because of reasons, as opposed to because of a shallow heuristic, or a potentially harmful bias.

Second, it avoids overselling the respective AI architectures. Considering the case of so-called reasoning LLMs can help. What the NLP community means with so-called reasoning LLMs is (1) that they generate so-called thinking tokens guided by a carefully designed prompt, and (2) that they have undergone some reinforcement tuning (Sutton and Barto, 2018), which combined should (3) lead to better performance at cognitively complex tasks, also called reasoning tasks, such as solving mathematical problems. However, what is not implied by calling an LLM a reasoning LLM is that its final verdict is in fact caused by a chain of reasons. Rather, the basic mechanism by which the “reasoning” LLM reaches its verdict is the same as the one by which a standard LLM reaches its verdict: next token prediction. The so-called thinking tokens generated simply provide a more robust base on which to draw this probabilistic inference. However, for a person not familiar with this use of “reasoning”, calling such an LLM a reasoning LLM seems misleading.

Thirdly, we suggest that this way of drawing the distinction nicely fits with an important desideratum of contemporary Language AI systems, namely explainability (Zhao et al., 2024). The black-box-nature of purely neural approaches is partly caused by the fact that the tokens LLMs generate in response to a question of the reasons causing a specific output are in fact not the real causes of the output: the reasons given are not causally connected to the output—it is not a *because* in this sense. In contrast, if you ask a typical adult human for her reason for believing in a claim, more often than not she will give you actual reasons, and she might even suspend belief in said claim if you can show her that her reasons are no good.

#### 2.2.1 Why NLP systems cannot engage in practical reasoning

Before discussing the kind of reasoning we think NLP Systems are in principle capable of, we briefly mention a kind of reasoning that we think is beyond them in principle: practical reasoning, that is, determining in a reasonable, rational way what one should do to live, ultimately, a good life. In such contexts, one can say that one has skin in the game: it matters for one's flourishing that one chooses wisely. Philosophers since antiquity have singled out rationality as the one feature that is characteristic of humans: We are said to be a specific sort of animal, namely a rational one. In a first approximation, rationality here comes down to responsiveness to reasons: Humans are not only acting based on their animal instincts, drives, and unconscious desires, but also in response to what they consider to be good reasons for an action, and ultimately to realize their idea of a good life: Aristotle held that the genuinely virtuous person is able to align her desires such that she holistically wills what she has recognized to be the right thing to do (see Aristotle, 1985; Halbig, 2021), whereas for Kant it is precisely a mark of rational autonomy that humans can do the right thing in spite of their animal desires pulling them in the opposite direction (Kant, 1999, B 434). As it seems misguided to assume that an LLM downloaded from an online repository and running on one GPU or another can have a good life in the relevant sense, it seems misguided as well to credit it with practical reasoning. It is simply the wrong kind of being for that.^6^

#### 2.2.2 Reasoning about formal-deductive validity: NLI and textual entailment

We here build on the survey by (Gubelmann et al. 2024). The modern task of Natural Language Inference (NLI) as a three-way classification problem (given two text passages, typically sentences, one of them called premise, the other called conclusion or hypothesis, the system must determine whether the label *entailment, contradiction*, or *neutral* applies) is rooted in the so-called RTE-challenges, from which they inherit a focus on deductive, and usually, but not exclusively, formal-deductive validity (Dagan et al., 2005; Bar Haim et al., 2006; Giampiccolo et al., 2007; Bentivogli et al., 2009). The most influential NLI datasets are probably, thanks to their sheer size of several 100 thousand pairs, the Stanford NLI dataset (Bowman et al., 2015) and the Multi-Genre NLI Dataset (Williams et al., 2018). Further, small NLI datasets are the so-called SICK dataset (Marelli et al., 2014) and the PPDB 2.0 (Pavlick et al., 2015), the latter being originally intended as a paraphrase dataset. We also note that the NLI datasets are not fully consistent in this regard. Compare (Gubelmann et al. 2023) for a study of the amount of non-assertions in MNLI (Williams et al., 2018). They find that, for instance, even though nothing follows deductively from a question, there still are questions as premises in MNLI.

#### 2.2.3 Reasoning about material validity

Within the realm of theoretical reasoning guided by material validity, so-called common-sense reasoning stands out. First, it has been a core task of AI in general and NLP in particular for decades, see (Brewka 1991) for an influential text from the nineties. Second, it has seen particular popularity in the past decade with the publication of a number of different challenge datasets, such as SWAG (Zellers et al., 2018), HellaSWAG (Zellers et al., 2019), PIQA (Bisk et al., 2020), and SIQA (Sap et al., 2019). Often, the goal of common-sense reasoning datasets is to assess whether the systems are able to fill in the blanks, what one simply is supposed to know. Consider Example (5) from the SWAG dataset (see also Gubelmann et al., 2024): In this example, we are supposed to infer that a woman that takes a seat at the piano is getting ready to play a concert, and hence that (d) is the only plausible continuation.

(5) On stage, a woman takes a seat at the piano. She (a) sits on a bench as her sister plays with the doll. (b) smiles with someone as the music plays. (c) is in the crowd, watching the dancers. d) nervously sets her fingers on the keys.

**Figure 3:**
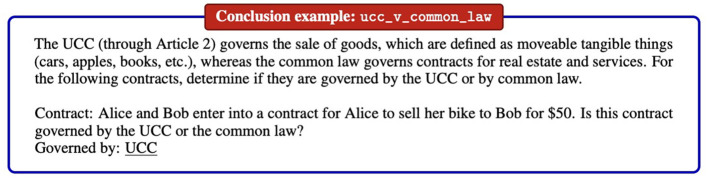
Example from LegalBench (Guha et al., 2023).

In addition to common-sense reasoning, there is a growing number of specialized subfields that require knowledge of specific material inferences. For instance, consider legal reasoning. Legal reasoning is important simply because it concerns a core domain of Western societies, the legal sphere. Legal specialization manifests in part in knowledge of what certain legal provisions materially entail. Important benchmarks are LexGLUE (Chalkidis et al., 2022), LegalBench (Guha et al., 2024), IL-TUR (Joshi et al., 2024), and LawInstruct (Niklaus et al., 2025). See Figure 3 for an example of the LegalBench dataset. Typical legal reasoning tasks are of this nature: They require knowledge of the subject matter, of core concepts, to determine the validity of material inferences. There are numerous domains like the legal domain, where specific knowledge of material inferences is relevant, e.g., mathematical reasoning, medical reasoning, or financial reasoning.

#### 2.2.4 Imitating reasoning and reasoning LLMs

Clearly separated from these species of inference-based reasoning is what we call quasi-reasoning: Processes and behaviors that prima facie appear to be processes of reasoning but which fall outside of the demarcation proposed here. Most importantly perhaps are systems and behaviors that imitate reasoning, but are not actually guided by genuine inferences. At root, the phenomenon is well-known to any teacher (or, for that matter, any parent): A student unconsciously follows a set of rules of thumbs, or heuristics, to solve a given task without really understanding it. For instance, she might pick up on certain regularities of exam question types to perform well at them without any genuine understanding of the subject matter. Less harmlessly, a headhunter might let her biases run wild and choose a candidate based on racial properties rather than actual reasons, such as relevant work experience. We emphasize that systems functioning in this way might perform well at certain reasoning tasks, such as NLI, but without actually reasoning, as they are guided by rules of thumb or bias rather than genuine reasons.

So-called reasoning LLMs, as mentioned above, Section 1, are characterized by generating so-called thinking tokens in addition to the actual output tokens to increase predictive accuracy at complex tasks. However, the basic structure of LLM inference, as sketched in the previous Section 2.1 remains intact: Rather than being guided by propositional reasons, the so-called reasoning LLMs predict, at any generation step, the token that is most probable given the previous tokens and its weights. Hence, it does not constitute a reasoning process proper as understood here.

**Figure 4:**
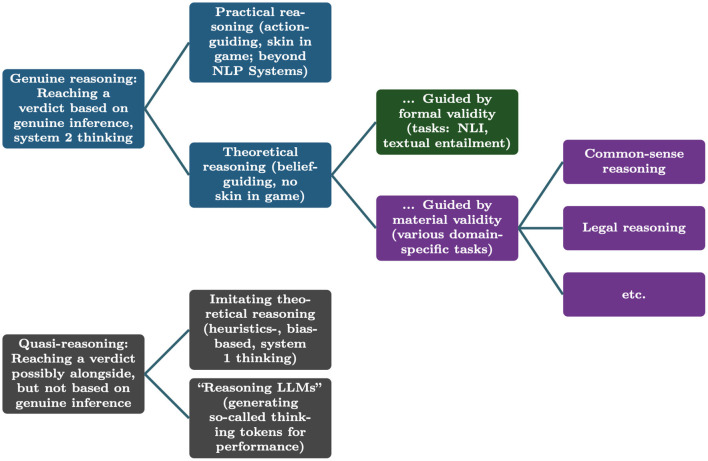
Overview: Important kinds of reasoning and instances of quasi-reasoning in contemporary NLP; green indicates modes of reasoning that the community is close to solving, violet modes of reasoning that it might solve in the near future.

We can summarize the systematic developed in this section in Figure 4. We re-emphasize that quasi-reasoning need not result in unreliable systems. However, given that these systems are not really guided by reasons, their performance can be hard to predict in the wild, as their behavior does not generalize along the real logical notions, but rather along their heuristics (which might work for the benchmark dataset but be useless in the wild).

## 3 The case for neuro-symbolic NLI

In this section, we will argue that neither purely symbolic nor purely neural systems have been able to make impressive progress toward Leibniz' calculus, and we will point out why Neuro-Symbolic methods have far more promise in this regard. We do so by first reviewing the empirical evidence in favor of our position and then grounding it in one of the leading theories of semantics and logic of our day.

### 3.1 The failure of purely symbolic systems

We do not think that too much evidence needs to be given to establish that purely symbolic systems did not show progress toward Leibniz's calculus ratiocinator. Indeed, a short comparison between the state of play before the last surge of neural methods [that is, approximately before the publication of the transformer architecture by (Vaswani et al. 2017), which built, among others, on the work of (Bahdanau et al. 2014)] shows that purely symbolic methods were simply too brittle in the wild.

At root, it is probably the inability of humans to make fully explicit the wealth of implicit knowledge that they effortlessly apply when navigating linguistic spaces: While they are categorically unable to enumerate explicitly all the meanings a word can have, they are excellent at implicitly surfacing the relevant meaning in a specific context. For instance, in the slightly modified Example (6), probably none of us has a problem understanding that *negative space with teeth* refers to panthers, and that, therefore, this inference is valid as well. However, we highly doubt that when writing a lexicon for the possible ways to refer to panthers, you would have thought of “negative space with teeth”.

(6) All panthers are mammals, and all mammals are mortal, therefore, all of these negative spaces with teeth are mortal as well.

This connects to the discussion in epistemology concerning the distinction between propositional knowledge, or knowing-that, and procedural knowledge, or knowing-how, see (Ryle 1945) for the founding work of this discussion and (Fantl 2008) for a recent overview on the debate. Unlike these epistemological discussions, which often center around rather exotic examples such as the so-called chicken-sexer who is able, with impressive precision, to identify the sex of newborn chicks, but who is categorically unable to name properties she uses in doing so, the present context gives very real grounding to the distinction. For instance, rule-based machine translation simply did not work because of the inability of system developers to make explicit even a fraction of the relevant meanings of words to translate. For a basic overview of the two machine translation paradigms, see (Bhattacharyya 2015), for evidence that rule-based machine translation did not work, just remember how poorly it functioned pre 2015. Note also that the transformer architecture (Vaswani et al., 2017), which currently completely dominates the NLP space, was introduced as a machine translation method.

### 3.2 The shortcomings of purely neural systems

While we assume that little argument is needed to convince the reader that purely symbolic systems did not deliver on Leibniz's dream, this might need some more effort regarding purely neural systems. To begin with, note that the focus of this study is on inference, including deductive inference, and not, say, on machine translation, where there is good evidence that this is a solved task for high-resource language pairs. We make our case in steps that follow the evolution of transformer-based reasoning methods.

#### 3.2.1 Fine-tuned encoder-only transformers

With encoder-only transformers, pioneered by BERT (Devlin et al., 2019), the task of NLI experienced a revival. There, it transpired that the purely neural approaches were facing the problem of generalization: They delivered excellent performance on data very similar to the one on which they were fine-tuned, but collapsed when confronted with the same task in the wild, or just with superficially different samples, see (Zhou and Bansal 2020), (Le Bras et al. 2020), (Utama et al. 2020), (Asael et al. 2021), (He et al. 2019), (Karimi Mahabadi et al. 2020), and (Bernardy and Chatzikyriakidis 2019).

As a consequence, the encoder-only transformers could clearly not solve NLI, and hence would not be able to deliver on Leibniz' vision of the calculus ratiocinator. In an influential study, (McCoy et al. 2019) conducted experiments to the conclusion that encoder-only transformers of the time picked up three kinds of syntactic heuristic at NLI tasks, namely lexical overlap, the subsequence, and the constituent heuristics. From the background of Section 2, this strongly suggests that these encoder-only transformers were simply guessing, that is, using a set of heuristics or bias instead of actually drawing inferences and engaging in genuine reasoning.

#### 3.2.2 Decoder-only LLMs

With the advent of large (and ever larger) decoder-only transformers, the scenery changed. The emergence of so-called Chain-of-Thought-Prompting, and the influence of one paper in particular, titled “Chain-of-Thought Prompting Elicits Reasoning in Large Language Models” (Wei et al., 2022), and with Zero-Shot reasoning (Kojima et al., 2022), especially around the year of 2022, there was genuine hope that a purely LLM-driven approach could deliver genuine reasoning, that is, reliable, reason-guided, generalizable inference capacities.

**Figure 5:**
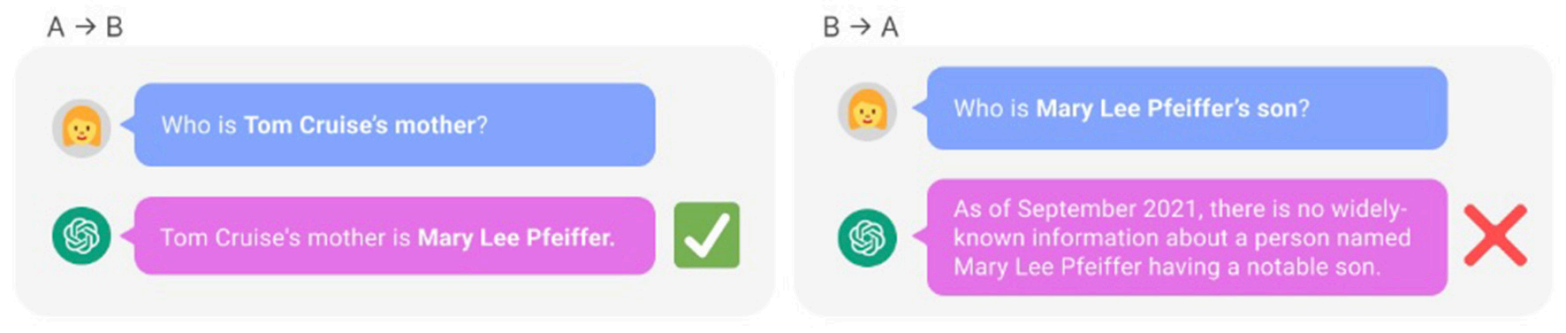
The reversal curse in action, the LLM tested was GPT-4 (Berglund et al., 2024).

However, later on, the enthusiasm was quenched by an increasing number of findings that seemed incompatible with this hope. For instance, (Berglund et al. 2024) demonstrated that LLMs that were able to correctly answer a relationship question in one direction where clueless in the other, which should not happen if the LLM had really mastered this kind of reasoning. For instance, GPT-4 was able to tell who Tom Cruise's mother was, but when asked who that mother's son was, it was clueless. See Figure 5 for an illustration from their paper.

Similarly, (Huang et al. 2024) demonstrated that LLMs are extremely bad at correcting their own mistakes in quite clear-cut cases. Overall, the picture emerges of LLMs as extremely able system-1-thinkers and as very good imitators of system-2-reasoners, but who reveal just more system-1-reasoning masquerading as system-2-thinking when properly probed.^7^

Currently, at the beginning of 2026, we seem to have left behind the next hype cycle, starting with the advent of so-called reasoning LLMs [see (Besta et al. 2025) for an overview and (DeepSeek-AI et al. 2025) for the pioneering work by Deepseek AI in early 2025], which raised hopes that we are now, finally, this time really, approaching purely neural systems that “genuinely” reason, that is, that behave in accordance with inferential rules over a large variety of problems and stably under adversarial probes. Unfortunately, in November 2025, a much noticed paper by (Shojaee et al. 2025) gave good evidence that even frontier reasoning LLMs drop in accuracy below 10% as the complexity of rather elementary reasoning problems posed to them increases just a little bit. Again, they where clueless where a typical human would not struggle to perform reliably.

In summary, it seems safe to say that the field has established beyond reasonable doubt that using extremely large LLMs trained with an amount of compute that would have been inconceivable just a couple of years ago is not the most efficient and effective way to get reliable reasoners. Furthermore, there seems to be solid, albeit not incontrovertible, evidence that the community will, as it were, bark up the wrong tree if it would continue to focus on purely neural methods. They are world-class guessers when it comes to NLI, but time and again have been shown to be just that: guessers instead of real reasoners.

### 3.3 The promises of neuro-symbolic NLI

After having argued that neither purely neural nor purely symbolic approaches to reasoning are able to deliver on Leibniz's vision of the universal calculus, or rather, of a pragmatically universal calculus, we will now present empirical as well as theoretical reasons to the conclusion that the combination of the two paradigms— neural and symbolic—has genuine potential of moving us toward the great vision.

At root, the idea is simple: Take the symbolic power of advanced logical calculi, as sketched in Section 1, and combine them with the world-class associative power of LLMs in the right way (on that note: the idea to refer to panthers as “negative spaces with teeth” is from Gemini, google's Chatbot).

We further specify this basic idea to the task of autoformalization, the task of accurately formalizing a natural language fragment into a formal language, typically Fregean first-order logic. Mastering this task is the last missing piece toward Leibniz' idea of the calculus ratiocinator, restricted to logical truths within the known principled limits: Once you have an accurate, meaning-preserving formal representation of the natural language text, you can indeed run an algorithm over it to assess whether the formalized version is logically true, that is, true under all interpretations of the atomic predicates and variables. This might not get you Leibniz's calculus directly and fully. However, it seems cleary a step in this direction.

(Olausson et al. 2023) were among the first to propose a Neuro-Symbolic Method that combined LLMs as candidate formal representation generators and symbolic solvers as checkers for these candidates to address the task of NLI, that is, to assess whether a given set of premises entails a given conclusion. (Dalal et al. 2024) even used tried and tested Prolog in place of a solver.

Intriguingly, last year, the community saw the publication of work that set an entirely new benchmark and justifies optimism that autoformalization might in fact be solved. (Quan et al. 2025) build on earlier research and develop a neuro-symbolic architecture for autoformalization in the NLI-subtask of explanation generation. It combines the associative, implicit strengths of LLMs (with deepseek-v3 proving to be best) with a symbolic theorem-proving framework called Isabelle (Nipkow and Klein, 2014) to achieve autoformalization precisions between 73% and 95% on key benchmarks, yielding double-digit autoformalization improvements across the board, sometimes reaching 40%. They achieve this based on two main tenets. (1) Harvesting the fruit of progress in both implicit, neural language modeling, epitomized by deepseek-v3, and highly sophisticated and versatile theorem-proving frameworks such as Isabelle. (2) By letting them interact in a way that near-ideally combines the strengths of both worlds. Thus, they invested considerable effort to optimize the feedback signal by Isabelle and its use by the LLM to improve the formalization.

Using this approach, (Quan et al. 2025) address the task of explanation generation for pairs of claims where one is known to entail the other, but where additional concepts and explanations need to be introduced to surface, or make explicit the deductive entailment. Their basic method then proceeds by letting LLMs generate good candidates to make explicit the implicit meaning aspects of premise and conclusion that are central for the validity of the inference. Then they use Isabelle to verify whether the current candidate explanation suffices to generate a deductively valid entailment. If it does, the method terminates, otherwise the LLM tries again, offering another candidate explanation and using the feedback from the solver in doing so. As mentioned, (Quan et al. 2025) achieve substantial improvements over their main competitor at this task, which is notably also already a Neuro-Symbolic approach. Purely neural approaches have not shown to be competitive. To get an idea of the kind of samples that the method solved, compare Example (7). There, the key is to understand that a violin is a kind of instrument, which allows to see the validity of the inference. The explanation that has been generated by the method supplies precisely this central piece of information (see Quan et al., 2025, p. 17748).

(7) **Premise**: A smiling woman is playing the violin in front of a turquoise background. **Hypothesis**: A woman is playing an instrument. **Explanation**: A violin is an instrument.

### 3.4 The theoretical case for neuro-symbolic NLI

In addition to the empirical evidence, can we provide any theoretical grounding for why it is precisely current Neuro-Symbolic NLP that might be able to approach a calculus ratiocinator? We suggest that inferentialist philosophy of language and logic can provide such a theoretical grounding. The specific variation of inferentialism that we will rely on has been pioneered by Robert Brandom [for his main works, see (Brandom 1994, 2010, 2021); (Hlobil and Brandom 2024)]. We highlight two aspects of this philosophy of language and logic. First, linguistic meaning is constituted primarily on the propositional level by the configuration of a network whose nodes are propositions and whose edges are entailment relations, the so-called implication space. From there, the meaning of concepts can be abstracted by the inferential role they play in propositions. For instance, a difference between *dog* and *cat* can be brought out as in Example (8).

(8) If Rudy is a cat, then he will not bark, but if he is a dog, and if his vocal tract is ok, then he will typically bark on occasions.

The second aspect of Brandomian inferentialism relevant for the current argument is logical expressivism, the idea that formal-logical analysis does not add any new truths or claims to the analysans, but merely surfaces, or makes explicit and systematizes the already existing structure. This presupposes that natural language text typically has a logical structure that can be made explicit.^8^

In this vein, one can say that the *characteristica universalis*, an artificial, ideal language that Leibniz was seeking for his *calculus*, and which he sketched on several occasions (Schneider, 1994) is simply natural language, properly formalized and systematized using a cleverly engineered Neuro-Symbolic Method.

Why then do we need LLMs if language is, at root, a system of inferentially connected claims and, derived from this, concepts-as-inferential-roles? In a word, because we are able to almost effortlessly navigate this network of inferences, but are unable to explicate this know-how into a know-that. Hence, the emerging methods for autoformalization are spot-on: They harness the full potential of progress made in calculating with formal languages by incorporating Isabelle, and they use LLMs as data-driven guessers of the relevant inferential connections that are needed. Taking up the distinction between system 1 and system 2 reasoning, one can say that the Neuro-Symbolic approaches sketched in the preceding section rely on system 2 thinking as much as possible and then fall back to LLM-driven system 1 thinking. The limitation is here not in the phenomenon, that is, language, but rather in our limitations of theorizing about the phenomenon, in other words, the limitation is epistemic: In our principled cognitive limitations of explicating the systematic rules in their entirety (which we usually effortlessly navigate implicitly).

So, from this Brandomian perspective, one can say that natural language, systematized and explicated into a modern formal language, can serve as Leibniz's *characteristica universalis* for logical truths within the known principled limits. Furthermore, given our cognitive limitations, we will need LLMs as smart, data-driven guessers of the meaning relations that are relevant for any inference that is deductively valid, but which is not directly formally provable due to implicit aspects of the meaning of the concepts involved (think of the negative space with teeth that should be interpreted as a panther, or of the violin that should be interpreted as an instrument).

## 4 Discussion and conclusion: make explicit what you can and leave the rest to LLMs

In this article, we have analyzed the concept of reasoning, as used in NLP/Language AI from a Leibnizian perspective, namely from Leibniz' vision of a *calculus ratiocinator*, a universal calculus that could derive the truth value of any proposition whatsoever. We suggest a concept of reasoning as essentially involving inference, where inference is understood as being guided by reason relations. From this perspective, when considering dual process theory, or system 1 vs. system 2 thinking, this means that only system 2 thinking constitutes genuine reasoning, whereas system 1 thinking amounts to heuristics- and bias-based guessing.

We have then surveyed the progress made at the task of Natural Language Inference (NLI). NLI is generally conceived as a three-way classification problem: Given two propositions, the method needs to determine whether one entails the other, or whether they contradict each other or are neutral. Solving NLI would achieve Leibniz' calculus for the domain of logical truths within the known principled limits. We have then surveyed the field of NLI, finding that neither purely symbolic nor (perhaps more controversially) purely neural approaches are promising in this regard. The former fail because we, as humans, are unable to make explicit all of the relevant meaning aspects of our concepts, and the latter fail because NLI is essentially a rule-governed, system 2 task, while LLMs are system 1 reasoners. However, we have also shown that very recent Neuro-Symbolic approaches show genuine promise to solve NLI. From the background of Brandomian inferentialism, this would be expected, as it essentially assumes that natural language, when properly systematized and explicated, can serve as Leibniz' envisioned *characteristica universalis*.

This suggests that current Neuro-Symbolic Methods in NLI are spot-on: They make explicit what we epistemically can (in the form of a formal logical calculus) and leave the rest – the fascinating, bewildering complexity of natural language meaning, which we masterfully navigate implicitly, but have failed to make symbolically explicit – to world-class guessers, namely LLMs.

We furthermore submit that, once reasoning guided by formally valid inferences has been solved, a similar combination of neural and symbolic elements could succeed at materially valid inferences. On our view, an agentic system, encompassing classical LLM generation modules as well as deep factual research abilities and a solver like Isabelle could also help to address material reasoning in a variety of domains, delivering something, albeit with some principled technical limitations, that Leibniz might have been dreaming about some 360 years ago.^9^

## Footnotes

1 We are very grateful to an anonymous reviewer for reminding us of these technical limitations.

2 One could also settle for a decidable fragment of first-order logic, say, the two-variable fragment, of which it was shown by (Mortimer 1975) that it is decidable.

3 In this article, “Language AI” and NLP are used interchangeably.

4 This distinction is modeled after the distinction between following a rule and acting in accordance with a rule, as it has been developed in the philosophy of language, see (Brandom 1994) and (Wittgenstein 1958). Note also that, on the view developed here, in line with (Burge 2010), consciousness of one's taking the premise to be a reason for the conclusion, sometimes called the "taking condition" after work by (Boghossian 2014), is not required. As will be detailed in the next section, all that is needed is a stable behavior along the right lines even in situations of adversarial probes.

5 We follow (Gubelmann et al., 2022) in our use of heuristic and bias.

6 In the same vein, we suggest that arguing should be distinguished from reasoning insofar as arguing leans much stronger into practical reasoning from the start, where context and practical wisdom are central. Reasoning, in contrast, has a *bona fide* theoretical branch. See (Gubelmann, 2024) for more details on this basically Kantian perspective on the possibility of LLM agency.

7 Adding to this, in a recent study examining a number of LLMs, (Gubelmann and Karray, 2025) find that they are unreliable at evaluating the validity or invalidity of rather simple syllogistic patterns.

8 This is opposed to both logicism, which maintains that the role of logic is to add new truths to our inventory, as well as to what we call a-rationalism, which maintains that natural language is too illogical and irrational for any coherent logical structure to be surfaced.

9 I am grateful for conversations with Marco Toscano and Andre Freitas who inspired me develop the main movements of argument in this paper.
